# Low-Cost GNSS Solution for Continuous Monitoring of Slope Instabilities Applied to Madonna Del Sasso Sanctuary (NW Italy)

**DOI:** 10.3390/s20010289

**Published:** 2020-01-04

**Authors:** Davide Notti, Alberto Cina, Ambrogio Manzino, Alessio Colombo, Iosif Horea Bendea, Paolo Mollo, Daniele Giordan

**Affiliations:** 1 Italian National Research Council, Research Institute for Geo-Hydrological Protection (CNR-IRPI), Strada delle Cacce 73, 10135 Torino, Italy; daniele.giordan@irpi.cnr.it; 2 Politecnico di Torino—DIATI, Corso Duca Degli Abruzzi, 24 10129 Torino, Italy; alberto.cina@polito.it (A.C.); ambrogio.manzino@polito.it (A.M.); iosif.bendea@polito.it (I.H.B.); 3 Dipartimento Tematico Geologia e Dissesto, ARPA Piemonte-Agenzia Regionale per la Protezione Ambientale, Via Pio VII, 9, 10135 Torino, Italy; alessio.colombo@arpa.piemonte.it; 4 CSP Innovazione nelle ICT s.c.a r.l., Strada del Lionetto 6, 10146 Torino, Italy; paolo.mollo@csp.it

**Keywords:** low-cost GNSS, warning threshold, unstable slope, cultural heritage

## Abstract

In recent years, the development of low-cost GNSS sensors allowed monitoring in a continuous way movement related to natural processes like landslides with increasing accuracy and limited efforts. In this work, we present the first results of an experimental low-cost GNSS continuous monitoring applied to an unstable slope affecting the Madonna del Sasso Sanctuary (NW Italy). The courtyard of Sanctuary is built on two unstable blocks delimited by a high cliff. Previous studies and non-continuous monitoring showed that blocks suffer a seasonal cycle of thermal expansion and a long-term trend to downslope a few millimeters (2/3) per year. The presence of a continuous monitoring solution could be an essential help to better understand the kinematics of unstable slope. Continuous monitoring could help to forecast a possible paroxysm phase that could end with a failure of the unstable area. The first year of experimental measurements shows a millimetric accuracy of low-cost GNSS, and the long-term trend is in agreement with other monitoring data. We also propose a methodological approach that considers the use of semi-automatized procedures for the identification of anomalous trends and a risk communication strategy. Pro and cons of the proposed methodology are also discussed.

## 1. Introduction

Landslides are one of the main natural hazards that can threaten the cultural heritage of humanity around the world [1,2]. The monitoring of unstable slopes joined with geological, geotechnical, and geomorphological studies is necessary to evaluate the risk and to plan the mitigation strategy.

Around the world, several archaeological/cultural sites were the object of landslide monitoring, for instance: Macchu Picchu in Perù [3], the Monemvasia historical site in Greece [4], and the Vardzia Byzantine monastery in Georgia [5]. In Italy, several cultural sites, potentially affected by a landslide, are monitored such as: the town of Pitigliano [6], San Leo [7], Orvieto [8], or San Fratello [9].

The choice of the best monitoring solution depends on several factors, like landslides’ typology and velocity, the interaction with anthropic structures, and the available budget [10,11,12]. Another essential element is the purpose of the adopted solution since the structure of a monitoring system used for civil protection activities is different from a monitoring solution adopted for a periodical control of the slope evolution. For this reason, a general solution that can be selected at every site and the condition is usually challenging to define. The site-specific characteristics thus are an important element that should be considered during the definition of the monitoring network and strategy.

Global navigation satellite system (GNSS) sensors are one of the well-known solutions for slope instabilities’ monitoring [13]. In recent years, the advance in GNSS technology allowed for creating new low-cost sensors that can provide continuous monitoring with good precision and accuracy and limited costs [14,15]. Nowadays, the use of low-cost solutions is commonly considered as not robust enough to support early-warning procedures. However, its deployment can be considered for permanent installations aimed at controlling the evolution of slope instability and detecting critical trends. In this context, these low-cost solutions can be regarded as a good alternative to periodical measurement campaigns. At the same time, low-cost instruments can help the installation of more precise and high-cost monitoring solutions only where and when it is necessary. As described in [16], the acquisition of monitoring data is the first essential step for the set-up of a decision-makers’ support procedure for risk management. Other important elements are a correct interpretation of monitoring data and a good communication strategy that can be used to share monitoring results with decision-makers and people that can be involved in the evolution of the slope instability. Correct risk communication is nowadays considered an essential element for a proper management of emergencies [17].

In this study, we present the first outcome of a continue and near-real-time monitoring network based on low-cost GNSS on the unstable cliff that affects the part of the adjacent area of the Madonna del Sasso Sanctuary, facing the Orta lake in Piemonte region (NW Italy). The sanctuary, built in the 18th-century, is an important cultural heritage site visited by many tourists and peregrines. The instability of the courtyard in front of the sanctuary is a factor of risk for people that visit this important cultural heritage site, but also for people that live at the base of the cliff, close to the Orta Lake. Different authors have studied the slope instability that affected the frontal part of the Sanctuary courtyard in recent years [18,19,20,21,22]. The Madonna del Sasso site is also monitored by the *Agenzia Regionale per la Protezione Ambiente—ARPA* (The regional environmental protection agency of Piemonte region).

In this work, the main feature of installed low-cost GNSS systems and the first outcome of the first-year monitoring are presented and discussed. We also propose a possible monitoring strategy that considers the use of semi-automatized procedures for the identification of anomalous trends. The strategy is part of a risk communication procedure based on the use of bulletins [16] and Operative Monographies [23].

## 2. Study Area and Past Monitoring Analysis

### 2.1. Geological Framework

**Figure 1:**
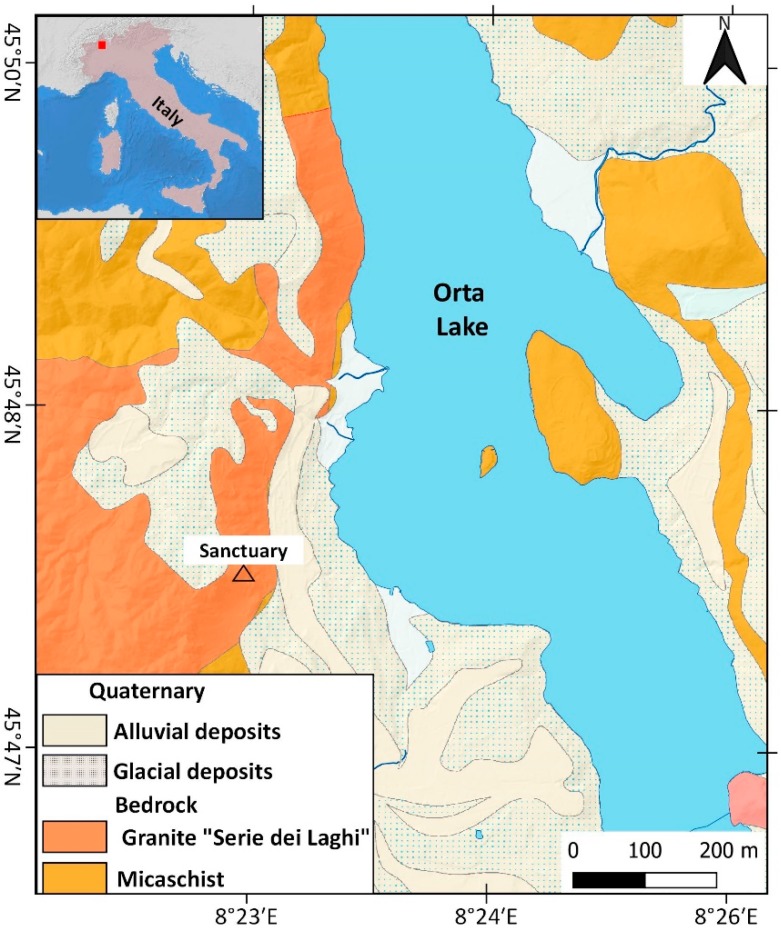
Location and geological settings of Madonna del Sasso site from the geological map of Piemonte retrieved from the WebGIS service of ARPA Piemonte and *Istituto di Geoscienze e Georisorse - Consiglio Nazionale delle Ricerche* CNR-IGG (Geosciences and Earth Resources Institute of National Research Council of Italy) [25], based on the work of [26].

The study area is located in NW Italy, on the western shore of the Orta Lake (Figure 1).

From a geological point of view, the sanctuary is located on a massive granite outcrop known as Granito di Alzo [24]. This unit belongs to the non-metamorphosed, and generally low deformed, granitic masses, related to a late-Hercynian magmatic intrusion (lower Permian). The magmatic unit outcrops along the contact between the lithologies of the “Serie dei Laghi” and the Ivrea-Verbano Zone. These granites, commonly known as “Graniti dei Laghi,” make a large batholith elongated in the NE–SW direction (Figure 1). The most widespread facies of the Alzo-Roccapietra pluton is a white, medium-grained biotitic granite. The pluton was not significantly tilted after the first intrusion and preserved its original position. The granite bedrock in which sanctuary was built shows excellent geomechanical features Geological strength index (GSI) >70 and high uniaxial compression strength >50 MPa), while the fractured blocks present poor quality [20].

**Figure 2:**
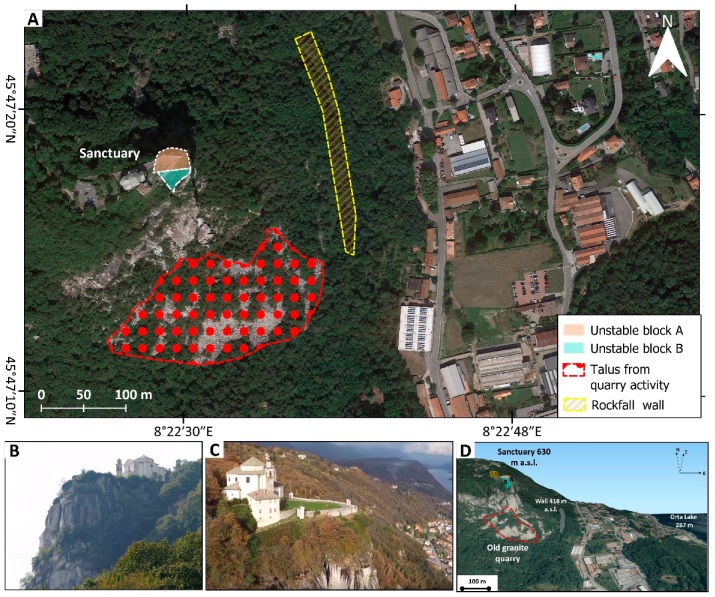
(**A**) shows an aerial image of the Sanctuary site in which are visible: (i) the location Sanctuary and the courtyard (ii) the ancient quarry deposits, (iii) a rockfall wall designed to protect the elements at risk; (**B**) and (**C**) report the view of the Sanctuary and of the unstable slope from North and South, respectively. The picture in panel (**D**) shows a 3D view from the South of the unstable cliff.

The Madonna del Sasso sanctuary is located on the top of a cliff at 650 m a.s.l. (Figure 2A) delimited by vertical slopes on three sides (N, E, and S) (Figure 2B,C). The scarps have a height of about 200 m. The cliff is characterized by the presence of different discontinuity sets that could create critical intersections, which are a predisposing factor for the activation of rockslides. The presence of this instability is quite evident in the frontal part of the courtyard, where one of the main discontinuity crosses from SE to NW the entire court.

The actual aspect of this cliff is also related to a combination of the structural settings and the presence of a quarry along the southern side of the slope that stopped its activity a few decades ago.

At the base of the cliff, talus and boulders are the result of past quarry activity and rockfalls. According to the critical stability condition and the presence of several elements at risk, at the end of the twentieth century, a protection wall was erected that aimed to reduce the possibility that detached blocks can reach the provincial roads and the nearby buildings (Figure 2D).

### 2.2. Structural and Geomechanical Characterization

Several studies were made on Madonna del Sasso site from the early 1990s when some evidence of instability appeared and the first countermeasures, like the rockfall wall, were realized.

**Figure 3:**
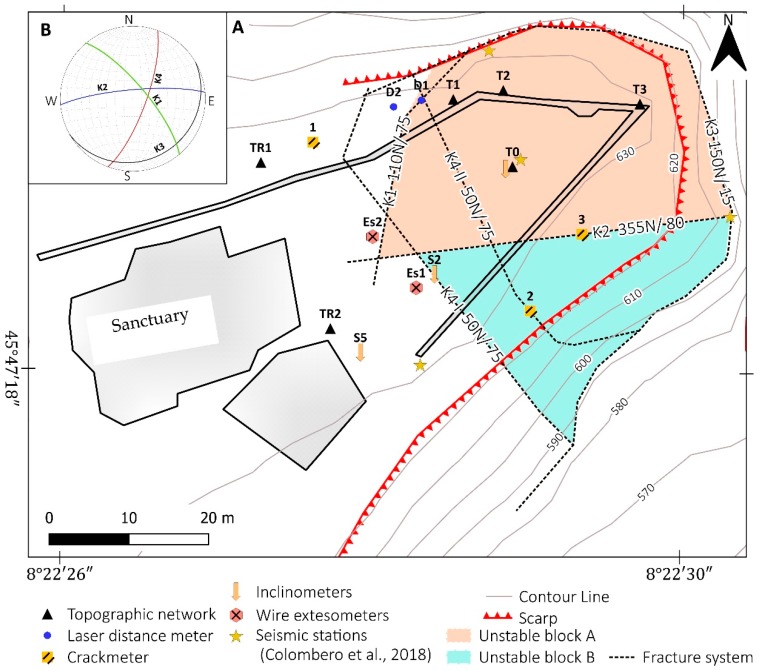
(**A**) detail of the fracture systems that limits the unstable blocks (**A**) and (**B**), based on results reported in [18,21]. The map also reports former and current monitoring systems installed by ARPA and Regione Piemonte on Madonna del Sasso site, together with seismic stations used by [27]; (**B**) shows the stereonet of the fracture system.

Lancellotta et al. [18,22] made a geomechanical characterization of the site. The detailed investigation carried out also using geophysical methods by Colombero et al. [20,21,27] allowed a good improvement of the knowledge of this case. In particular, Colombero et al. [21] identified an open and pervasive fractures system delimiting two prone-to-fall sectors (A and B) having estimated volumes of 6000 and 6300 m^3^, respectively (Figure 3). The two sectors are separated by the sub-vertical fracture K2 (355 dip direction/80 dip) having an approximate open depth of 15 m and are truncated at the base by the low-dipping fracture K3 (153/15). Fractures K1 (113/65) and K4 (52/80) (Figure 3B) separate the back of the two compartments from the stable cliff. In particular, fracture K4 shows an evident displacement step on the panoramic square located at the top of the cliff. The investigation found that the fracture is open up to a depth of about 16 m [21].

The studies of Colombero also allowed the identification of quite low seismic velocities in the whole unstable sector that suggest the probable widespread presence of dry cracks and secondary fractures [20].

### 2.3. Unstable Slope Behavior

The monitoring system installed by Regione Piemonte and ARPA since 1990 has been composed of several instruments like inclinometers, laser distance-meters, and topographic benchmarks network [19]. Most of these monitoring activities have been performed by limited periods. Wire-strain gauges collected data only for a short period and were used to understand the thermal behavior of rock mass and fractures systems (Figure 3). In the Colombero study [22], the geo-structural study was combined with borehole seismic and micro-seismic (MS) monitoring campaigns. The authors also used a detailed analysis of topographic monitoring data of ARPA Piemonte to understand the rock mass evolution and fracture system behavior. The most important results of Colombero studies are:

1. The observation of a seasonal cycle of thermal expansion of the rock mass. This interesting behavior was detected by combining MS investigation, topographic monitoring, and wire-strain gauge measurements. The MS rate was coherently recognized to be mainly driven by temperature. The maximum frequency of events occurred during summer, with thermal expansion, while a minimum of the MS events was recorded in winter months when the fractures are opening due to contraction on the bedrock.
2. The MS investigation pointed out the presence of many MS events, which are related to rapid air temperature variations (temporal gradient), or marked temperature differences between the cliff’s faces (spatial gradient). These events could cause differential thermal dilation and induce thermal stresses leading to microcracking processes within the cliff, as already demonstrated by [28].
3. The temperature control was found to be the cause not only of fracture opening/closing with a seasonal frequency, but also for a general change in the vibration directions measured during MS investigations of the two unstable blocks. In addition, the daily cycle of thermal variation influences unstable blocks behavior as observed on seismic noise resonance [22].
4. The observation of a long-term trend in which block “A” tends to slide towards NE of 2/3 mm per year. This trend is probably related to progressive weakness and degradation of rock bridges in fractures caused by the expansion–contraction cycle.

**Figure 4:**
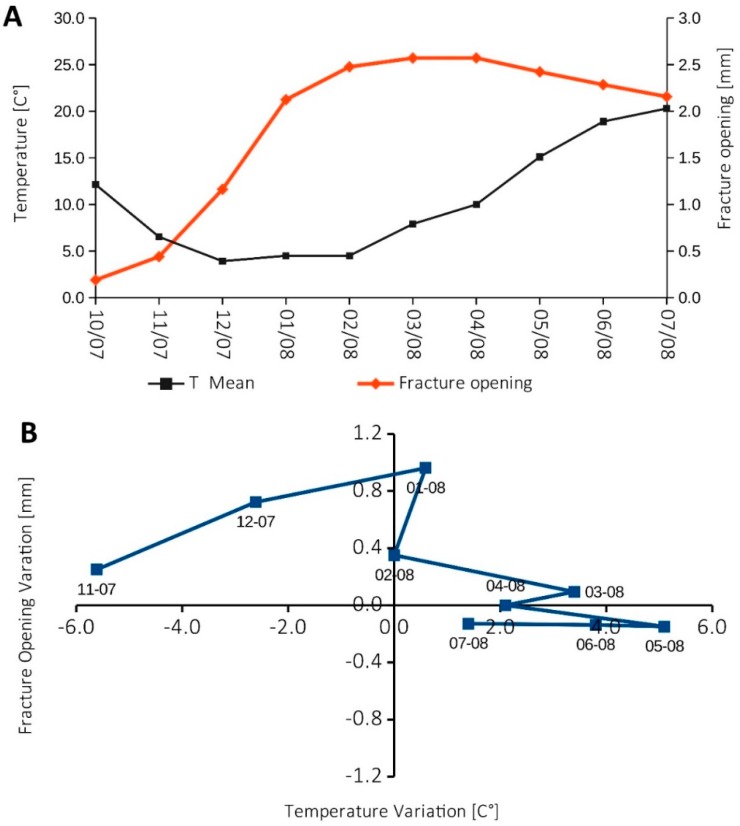
(**A**) shows the monthly averaged temperature [°C] and fracture opening [mm] from October 2007 to July 2008; (**B**) reports a plot of the variation of the fracture opening [mm] as a function of the temperature variation [°C], calculated on a monthly temporal scale for the period October 2007–June 2008; data source: wire extensometers of ARPA Piemonte.

In addition, past displacement monitoring campaigns (2007–2008) at the wire extensometers across fracture K4 highlighted a partially reversible seasonal fluctuation of fracture opening, driven by the air temperature (Figure 4A). This trend is visible in Figure 4B, where the change of air temperature is compared with the changes in fracture opening from October 2007 to July 2008. Maximum fracture opening was recorded during winter months (up to 1 mm/month), likely due to the rock mass thermal contraction; while a slight closure (about 0.2 mm/month) was registered in summer as a result of the rock mass thermal expansion.

Colombero et al. [27] also suggested to install a continuous topographic monitoring system, joined with MS noise measurement on the site, in order to better define the seasonal cycle of thermal dilation and the long-term cycle of degradation and slide of the blocks. According to this suggestion, this site has been chosen for the development of the presented low-cost GNSS monitoring network.

## 3. Materials and Methods

The work made on Madonna del Sasso site was divided into four main packages:

1. The production of an operative monography (OM). In this project, we decided to adopt the OM approach [23] for the acquisition and organization of all available information (e.g., monitoring data, thematic studies, publications, and maps). The first step of the project has been the collection, analysis, and organization of the available material. This approach is particularly useful when the analyzed site has a long history of studies, in situ exploration, and monitoring that could have produced a large amount of data that is not always well organized. The OM has been very useful for the definition of what has been already defined by the previous study. Thus, we used the collected literature data for the identification of better positions for GNSS installation.
2. The re-analysis of ARPA-Piemonte topographic network data. The topographic dataset acquired by ARPA Piemonte is available from 2006, and it represents the most important source of information for the definition of displacement trends. In the re-analysis, we removed some outlier measurements, and we recalculate linear regression of displacement time series (horizontal and 3D). This helped the comparison with other monitoring instruments like the PS InSAR and the new GNSS data.
3. Design, implementation, and test of GNSS low-cost sensors. The GNSS system has been developed and tested in the laboratory. After these first phases, the system has been installed in the unstable area, and it becomes the first in place continuous topographic monitoring solution adopted for the study of the evolution of the Madonna del Sasso cliff. Two low-cost GNSS sensors were installed on-site to measure the position of two different representative points continuously. The adopted monitoring network is equipped with hardware and software solutions that guarantee not only the in-situ acquisition but also the raw data transmission and processing.
4. Definition of a semi-automatic early warning (EW) procedure. The final step of the project has been the development of a GNSS results analysis aimed to implement an early warning (EW) procedure. The EW procedure is a step-by-step methodology that has the aim to firstly define critical alert thresholds indicative of a possible increase of blocks instability. After validation, the alert could be used to issue a bulletin for risk communications [29].

### 3.1. The Operative Monography (OM)

As presented and discussed in [23], one of the most critical elements that often characterized slope instabilities monitored or studied for a long time is the availability of many data without a good organization and representation of obtained results. Very often, data have been acquired by different working groups and preserved by different private companies or administrations. A practical (and also standardized) organization of what we already know about the studied phenomenon is the first fundamental step for better management of a possible emergency related to an increase of the activity of the slope instability. Practical standardized documentation also helps for the identification of possible weak points in the state of the art of available studies.

As mentioned before, the Madonna del Sasso case study has been studied by several research groups, and now is one of the monitored slope instability in the Piemonte Region. The presence of many studies, projects, risk assessment analyses and remedial works means the presence of a large amount of data and information often distributed in different local or regional administrations or research centers. The ARPA Piemonte collected part of the information in a system called SIFRAP.

The SIFRAP (Sistema informativo fenomeni franosi in Piemonte–information System of Piemonte region landslides) database (in Italian). This system is composed of cartographic information (organized using a web GIS platform) and different forms with a progressive level of detail. In particular, SIFRAP forms are organized in three different levels of detail: (i) level one—a synthetic description of the landslide according to with the IFFI Project, the Italian landslide inventory [30]; (ii) level two—composed by level one and a more detailed description of available information and a dedicated geomorphological map of the slope instability; (iii) the highest level that is available only for few landslides in Piemonte and that provides a detailed study of the slope instability. For Madonna del Sasso, the SIFRAP system has a second level form available (http://webgis.arpa.piemonte.it/Web22/sifrap/ii_livelli/103-01641-00.pdf). Here, SIFRAP gives a good representation of the case study, but a large amount of information acquired in the last three decades is not adequately represented.

The OM facilitates the assessment of the state of knowledge, which is a necessary step for a complete comprehension of each phenomenon. The document is addressed to end-users like civil protection, researchers, technicians, and local authorities. In the case of Madonna del Sasso, we collected the information coming from Lancellotta and Colombero studies.

The OM contains a chapter focused on resuming the main monitoring result of ARPA and Regione Piemonte as well as the interpretation of MS monitoring results published by Colombero. The outcomes of the new GNSS monitoring system are added in a chapter dedicated to “further studies in progress” that can be progressively updated on the base of new monitoring and study results.

The use of OM has been very useful for the collection and organization of available material that has been the base for the GNSS monitoring system development and installation. Part of the OM results are synthesized in the description of the Madonna del Sasso case study and the chapter dedicated to the topographic monitoring result analysis.

### 3.2. Available Topographic Monitoring: Data Analysis

The most important dataset comes from topographic measurements (active from 2006) and laser distance meters (active from 2009) made and collected by ARPA Piemonte. All active monitoring instruments are installed only on block A (Figure 3). ARPA installed four topographic benchmarks at this site: three are located on the parapet of the courtyard (T1, T2, and T3), and one in the unstable sector of the courtyard (T0). Two adjunctive benchmarks are located in a stable area near the sanctuary and they are used as reference points. The benchmarks have been measured about 2–3 times per year since 2006. The two laser distance meters are located across the K4 fracture on the north side of the courtyard, and they provide about two measurements per year since 2009.

Available satellite Interferometric Synthetic Aperture Radar (InSAR) data are not very significant. On the unstable blocks, only one persistent scatterer (PS) of the Radarsat satellite (ascending geometry, Omegna dataset) provided by ARPA Piemonte is available and covers the period (2003–2009). However, the PS InSAR was taken into consideration because the estimation of displacement rate on the long-term is quite accurate (+/−2 mm/year). In addition, to improve the accuracy of the measure, we used a PS located in the nearby stable area as a local benchmark. Available monitoring data (Table 1) have been re-processed. The re-processing of the data consisted into recalculating for each type of data (topographic, Laser distance meter and InSAR) the linear regression of displacement for a better comparison of velocity and time series. The results of monitoring analysis were used firstly to choose the best place to install the new GNSS. In a further phase, monitoring data of ARPA Piemonte were used for validation/comparison of GNSS data for the first period of measurement (December 2018–May 2019).

### 3.3. Design, Implementation, and Installation of a Low-Cost GNSS Monitoring System

The previous research [15] showed that, even with mass-market receivers, it was possible to detect displacements imposed up to five millimeters in planimetry and one centimeter in altimetry. In the previous study, the best results were achieved with an uBlox EVK7T (Thalwil, Switzerland) receiver, a Garmin GA38 (Olathe, KS, USA) mass-market antenna and when the distance from the master station was about few hundred meters. The phase ambiguity fixing was performed with the use of the L1 GPS frequency only [15].

The instrumentation installed on the Madonna del Sasso sites involved the use of mass-market uBlox EVK8T receivers that provide data on the first frequency of the three GNSS constellations: GPS, Galileo and Beidou. It also based on the Garmin GA38 antenna able to trace these frequencies and a distance from the nearest permanent station of less than 10 km. A short distance between master and rover makes it possible to neglect the tropospheric and ionospheric residues in the double phase differences, and it has almost always been possible to integer fix whole-phase ambiguities with multiple constellations and one frequency.

In the next paragraphs, we better describe the instrument installed on the site and the GNSS data processing.

#### 3.3.1. Hardware Technology Installed and Calibration

**Figure 5:**
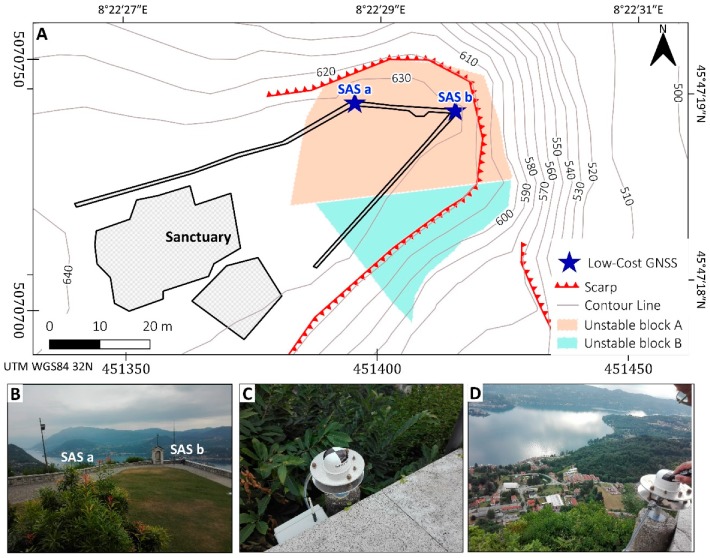
(**A**) shows the location on maps of Global Navigation Satellite System GNSS antennas “SAS a” and “SAS b” with latitude-longitude (upper right) and UTM WGS 84 (bottom left) coordinates; (**B**) a picture shows the location of antennas from the courtyard perspective; (**C**) the photo shows a detail of “SAS a” antenna [451401.968 E, 5070726.618 N, 682.378 h–WGS4 32 N]; (**D**) shows a detail of “SAS b” antenna SAS b [451384.123 E, 5070731.990 N, 683.187 h–WGS4 32 N].

**Figure 6:**
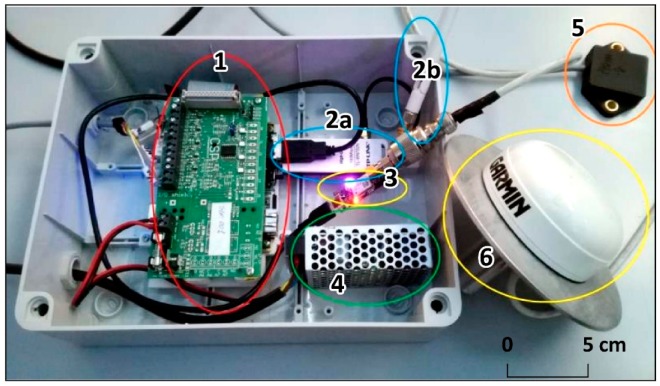
The prototypal multisensor box and the Garmin Antenna. 1 = Micro PC mainboard, 2a = Dongle Wi-fi and relative antenna (2b), 3 = GNSS mass-market uBlox EVK8T Receivers, 4 = Power supply, 5 = Tri-axial accelerometer + temperature sensor, 6 = Garmin antenna.

In Figure 5, the location of the instruments (SAS a and SAS b) in the courtyard of the Sanctuary is shown. The set of instruments of the prototypal multisensor box installed the monitoring sites can be seen in Figure 6. The main components are:

1. A micro PC with a Linux operating system that controls all the sensors. It is equipped with a mainboard (1 in Figure 6), four powered USB interfaces, a removable memory card on which the operating system and data are stored. The micro PC is programmable by remote control like (e.g., via Dongle Wi-Fi, 2a and 2b in Figure 6) by any Linux computer. The micro PC also manages the dialogue with the uBlox receiver (3 in Figure 6), with the accelerometer and the temperature sensor. The system must be externally powered either by a power supply (4 in Figure 6) or by a solar panel. The micro PC also manages the transmission of data to the control center, both the accelerometric (5 in Figure 6) and temperature data and the corrections in the Radio Technical Commission for Maritime Services (RTCM) format of the GNSS sensor.
2. uBlox EVK8T GNSS receiver (3 in Figure 6). It provides data on the first frequency of the three GNSS constellations: GPS, Galileo and Beidou. However, during the months in which the receivers provided their data, the Beidou constellation did not yet have a sufficient number of satellites to improve the robustness of the positioning. The Galileo constellation, which is more visible in Europe, although not yet complete, has for now only provided help in fixing the GPS phase ambiguities.
3. Garmin GA38 mass-market antenna (6 in Figure 6). As the mass market antennas have been mounted on a specially constructed aluminum plate that forms the circular ground plane. A second plate was attached to this plate with a spacer and finally a pin with a 5/8 thread that allows the antenna to be mounted on any type of topographic base. Before installing the antennas, precision measurements were carried out to calculate the position of the phase center with respect to the bottom of antenna mount but also with respect to the top of the small dome of each antenna. For deformation monitoring, this operation is not mandatory as only relative movements are required. It was useful both because the “zero” position was calculated with high precision with geodetic antennas and receivers, and because the dome was painted in a checkerboard pattern to be observed photogrammetrically. The coordinates of the center of the dome have been used and will, therefore, be used as a control point in these reliefs (the Ground Sample Distance–GSD-is about 6 mm). The position of the phase center was obtained with an accuracy of one mm utilizing simultaneous measurements with a geodetic receiver and calibrated antenna, using the “antenna swapping” technique. The planimetric phase center coincides with such precision with the physical center of the antenna.
4. A triaxial accelerometer “4030” (5 in Figure 6) from TE Connectivity^®^, usually used for structural monitoring was mounted on the bottom of the ground plane under the GNSS antenna. This sensor, with a range of ±2 g, provides an mV output, which can be converted into acceleration values and relative angular values. The residual noise is an angular value of 0.022° (0.38 mrad or 3.8 mm@10 m). The monitoring of angular values with acceleration measurements is theoretically independent of time, not being linked to integration operations as in the case of gyroscopes. This makes the accelerometric angular measurement independent of the acquisition measurement duration. The accelerometers were mounted on the lower part of the antenna ground using a compass to orient them and after having made this plane perfectly horizontal. Each sensor, before being installed, has been individually calibrated in the laboratory with the appropriate matrix described in [31]. After calibration, the accuracy detected by the laboratory is 0.02°, about 10 times better than what would occur without calibration. After the examination of six “4030” accelerometers calibration constant, it was found that the calibration values differ from each other considerably: it is therefore not possible to perform an average calibration for this family of sensors that must be individually calibrated.
5. Temperature probe. It has been installed near the accelerometer, below the ground. Temperature is transmitted together with the accelerometric data to the control center. During the measurements performed in the laboratory, there was no particular relationship between the accelerometric and temperature measurements.

The cost of installed instrumentation is about €500 (Table 2). The installation cost is very dependent on the characteristics of the site, its accessibility, and the existing infrastructures (electricity grid, data transmission network). Under normal conditions, the cost is empirically estimated at about €500.

#### 3.3.2. GNSS Data Processing

The processing of data from low-cost sensors took place using open source software from the “RTKLIB” library (http://www.rtklib.com). This software is installed both inside the minicomputer that transmits the RTCM stream of the uBlox receiver, and at the control center.

In the control center, the STR2STR program stores the RTCM flow received every second from the monitoring stations, in a data file packaged both hourly and daily. Even the automatic GNSS data processing techniques mainly refer to a series of RTKLIB free-use package programs as well as to some software, specifically built and necessary for these purposes, aimed at scheduling a different operation:

1. Automatic data acquisition from Ublox single-frequency receivers;
2. Daily packaging of these data according to standard RINEX formats;
3. Data acquisition of real or virtual master stations for post-processing;
4. Automatic post-processing of the rover stations with master stations or with virtual stations for movement monitoring;
5. Post-processing of the data of stations close to each other, for deformation monitoring;
6. Automatic sending of a reasoned report of the post-processing results both via email and to web platform;
7. Construction of graphs of displacements and deformations and sending it specialized technicians to interpret the results.

Another topic is the real-time transmission of data. Even if post-processing involves daily processing data, for which acquisition rates of 30 s are sufficient, data transmission in the RTCM 3.01 format at a rate of 1 s does not require special equipment. This can allow for post-processing as well as future real-time control. These files in RTCM format every hour and every day are converted into a RINEX format measurement file through the “CONVBIN” command. Finally, it is necessary to treat this data with the same periodicity; this is possible through the “RNX2RTKP” command. However, differential post-processing requires the use of data from at least one master station. For the stations of Madonna del Sasso, the master station is the permanent station of Gozzano, about 9 km away. At the end of the conversion, and the differential processing in post-processing, the RINEX observation and navigation files must be compressed and archived. The results of the differential positioning come together in ASCII files. Finally, a file that contains a summary of the results of all daily treatments for all stations and for any treatment is created. Processing is considered to be failed if the complete fixing of the phase ambiguities, and the ratio test [32] is not achieved.

Finally, a report is sent by e-mail of the daily post-processing outputs. This report is sent to people with expertise in GNSS processing and in landslide monitoring. These outputs and the statistical controls are also deposited in the cloud, together with the graphic drawings and the hourly processing reports.

Alert information is provided through a table containing codes similar to traffic light signals, which will be described later.

All these commands are scheduled on the computer and are completely automatic.

### 3.4. A Methodology for the Assessment of Warning Thresholds

**Figure 7:**
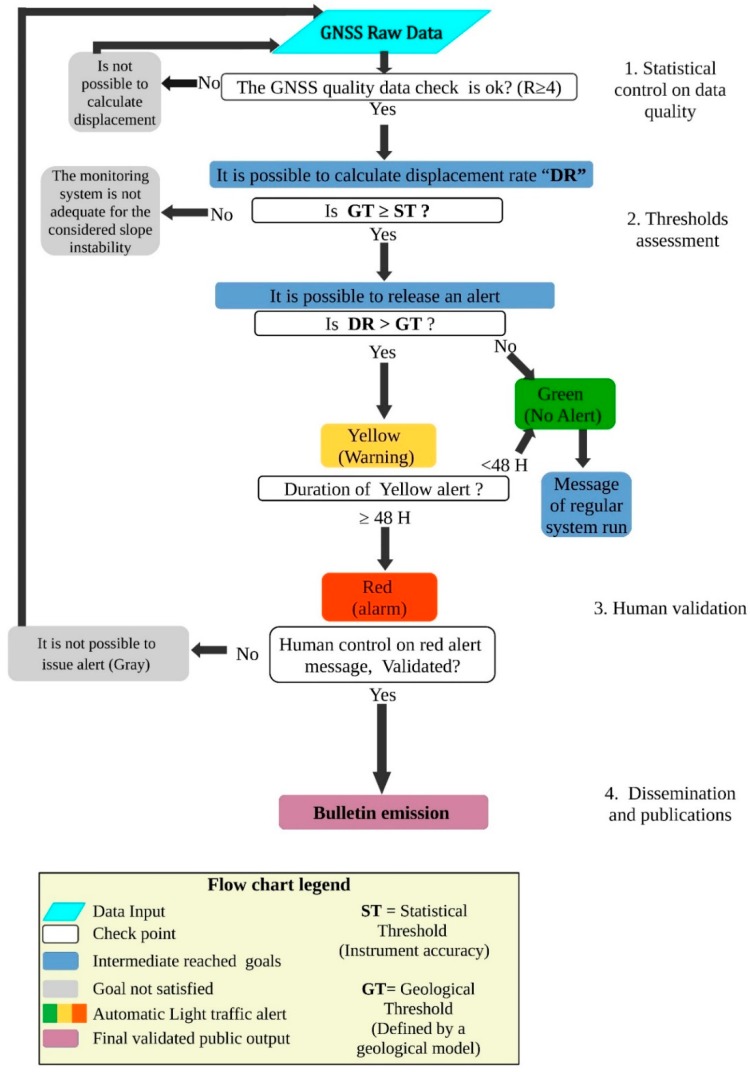
Flowchart of the proposed procedure to define and test the alert threshold based on continuous and low-cost GNSS monitoring. The chart is focused on daily processing, but the same schema could be applied to long-term thresholds.

After the setup of the GNSS, we also improved a methodology for effective use of GNSS measurements. The developed system divided in a sequence of steps that starts from the acquisition and processing of data and ends with a dedicated dissemination procedure of obtained results. The flowchart of Figure 7 shows the proposed procedure that, in this case, it is focused on GNSS data, but it could be applied to a generic landslide monitoring system. The main steps of the monitoring management procedure are:

(i) The statistical control of raw data. We used an algorithm that detects signal-noise ratio, called a Ratio test (R), developed by [32]. Only raw data with a minimum acceptable quality (empirically fixed at R ≥ 4) pass to the further phase of processing. If the raw data have an R < 4 (low quality), no further elaborations are possible and the procedure starts again with new data.

(ii) The thresholds assessment. In this step, an automatic algorithm checks if the movement overpasses the statistical and the geological thresholds. The statistical threshold (ST) is related to the accuracy of the instrument: in our case, it is twice the standard deviation (2σ) around the value foreseen by the regression line. The geological threshold (GT) is based on the geological model, and it is related to the movement rate that, according to expert studies, could indicate an incipient paroxysm phase. It is important to remark that the GT must be higher, or at least equal, than the ST. If the model shows that the GT is below instrument accuracy, probably our monitoring system is not adequate for the considered slope instability. In the case of Madonna del Sasso, the GT for short-term displacement was not yet determined, but we can consider that it can be very close to the ST. According to long-term monitoring results, the long-term threshold was empirically fixed at 3 mm/100 days (or 11 mm per year). This threshold has been defined considering the analysis of long (2006–2019) topographic monitoring sequence. On short period, measurements that overcome the ST have to be carefully considered to check if this result can be considered a spike or the beginning of a critical phase. To solve this problem, the system required a double validation before declare and alarm: (i) firstly, the monitoring result must be statistically validated (R > 4); (ii) then, it should be analyzed and also validated from the geological point of view.

**Figure 8:**
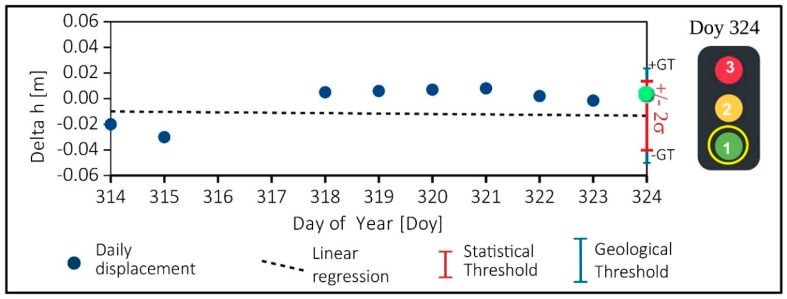
An example, using a simulated dataset, of daily solution for vertical displacement (Delta h). In the Day of Year 324, the displacement (green dot) is an insight into the statistical threshold (±2σ on the regression line) and below the geological threshold (GT). This means that normal conditions (green light–flag = 1). The days 316 and 317 have no measure because the ratio test was <4.

If the displacement is statically validated, an automatic warning report is daily generated: the procedure considers, as usual, three levels of warning:

1. Greenlight that means regular conditions (i.e., the ST and GT are not overpassed (Figure 8 and Table 3);
2. Yellow light (warning level), if the GT has been overcome for one day (≥24 h). In this phase, we can also consider the possibility that it could be an error in the system and, for this reason, we have to wait for the second cycle of measurement to obtain a validation of the trend,
3. Red light (alarm level) if the GT has been overcome for consecutive two days (≥48 h). In this condition, monitoring results should also be evaluated and validated from the geological point of view. This procedure has been implemented to be totally automatized and requires the human check only at the end of a quality control procedure, for the last validation of the alert.

(iii) The validation of the alert. This is a human-based procedure in which experts, on the base of automatic alert messages that coming from the monitoring systems, decide the congruency of the alert messages and they activate alert communication procedure to the authorities and the public. This step can be done using a single monitoring system, but a complex monitoring network based on redundant instruments is usually suggested. In this phase, the contextual use of data from the monitoring system and the OM can support decision-makers in the evaluation of the level of emergency and the definition of risk mitigation activities.

(iv) The alert bulletin dissemination. If the alert has been validated, the implemented system can update a dissemination bulletin aimed to inform the population about the last monitoring results and the level of activity of the considered phenomenon. According to [29], this bulletin can be redacted manually or automatically from a pre-defined model adapted to the monitored phenomenon. With the proposed approach, the bulletin can be disseminated only when a robust monitoring system supports an alert, and monitoring results have been geologically validated.

## 4. Results

### 4.1. Monitoring Data Processing

**Figure 9:**
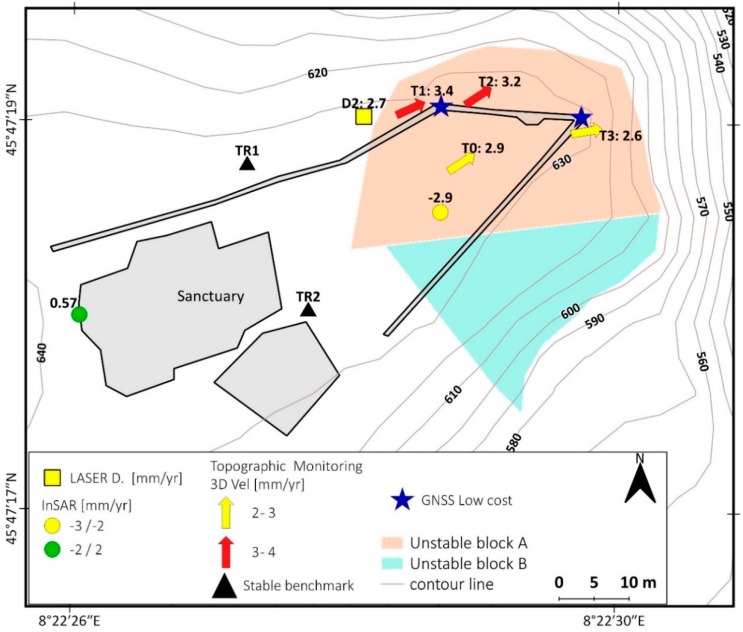
Displacement rate map measured by ARPA Piemonte instruments: laser distance-meters (period 2009–2018), InSAR (2003–2009), and the topographic network (2006–2019). The map also shows the location of the new low-cost GNSS systems.

**Figure 10:**
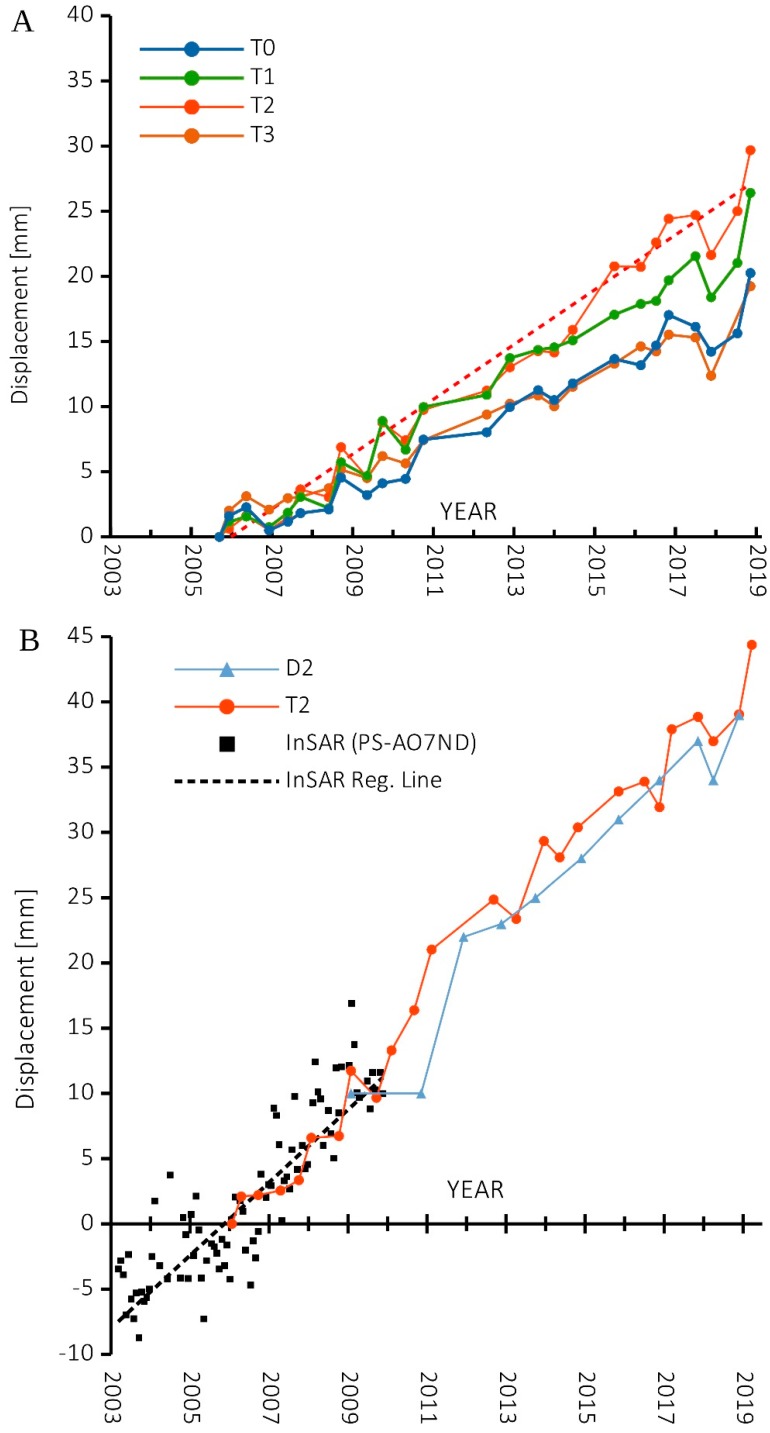
(**A**) shows the Time Series of 2D (horizontal) displacement of the topographic benchmarks (T0, T1, T2, T3) and the linear regression represented as red dashed line for T2 (the equation is f(x) = 0.006x − 224.3; R^2^ = 0.97; (**B**) shows the 3D time-series displacement of: benchmark T2; the laser distance meters D2; the PS InSAR data (PS id: AO7ND Radarsat ascending dataset) with its linear regression represented by the black dashed line (the equation is f_(x)_ = 0.008x − 295.3; R^2^ = 0.77).

In this chapter, we present the results of the topographic monitoring system installed by ARPA Piemonte from 2006. The monitoring network is composed of four topographic benchmarks and two laser distance meters that are periodically measured (up to 3/4 measurements per year) (Figure 9). The results of laser distance meters and topographic benchmarks are in agreement and show the long-term movement trend of block A toward NE, with a rate of 1/2 mm/year. We also consider the vertical displacement (downward) and the full displacement rate is 2/4 mm/year (Table 4) that are in good agreement with available Persistent Scatterer (PS) InSAR data of the Radarsat satellite (10 B). The time series of topographic data seems to show seasonal oscillations that are in agreement with the documented seasonal cycle: measurements made after the winter show higher displacement then summer measurements. However, due to the few and not constant measurements per year and the limited displacement rate, it is hard to validate the seasonal oscillation because the entity of the oscillation is very close to the signal/noise limit (Figure 10A).

### 4.2. GNSS Data Processing: Quality and Reliability

#### 4.2.1. Alert Assessment with Short-Term Displacement

As described in the previous Section 3.3.2, it was decided to analyze only the solutions with the ratio test [32] higher than or equal to four. We do not also analyze the solutions to unfixed ambiguity or the results that show the absence of daily data in these stations. These filters allow us to have more statistically robust results.

On this subset of data, an estimation of the median absolute deviation (MAD) [33] type was performed for the calculation of the regression line. This analysis occurs if the data of the day just passed are present, but the parameters are calculated without using the latter data. The same treatment takes place with hourly data. With the estimate of the regression line, for each component, the measure of the coordinate of the last epoch, that is of the day or the hour just passed, is foreseen. The estimate also calculates the value of the standard deviation of the data. The current position is considered acceptable and without alarms, if the coordinates are in the range of twice the standard deviation (2σ) around the value foreseen by the regression line, without the use of the last measurement themselves (Figure 7).

In the case of Madonna del Sasso, as mentioned before, was not possible to assess a GT, so we analyzed the results only from a statistical point of view, assuming that GT is close to ST (see Section 3.4). The results of five months of monitoring are resumed in Table 5.

When the raw data showed low quality (R < 4), it was not possible to calculate the displacement rate and no alarm could be issued (code 0). In our case study, this happened in the 22% of the period for GNSS “SAS a”, mainly for logistics or maintenance problems (e.g., lightning strike) to the experimental system.

When there are data to be examined, we have followed the procedure described in Section 3.4 (Figure 7 and Figure 8). We obtained for GNSS “SAS a” the following results:

1. “green-light” or normal conditions (code 1), happened for about 98.5% of the valid measure,
2. “yellow-light” or attention level (code 2), happened only for 1.5% (two days over the entire periods) of valid data both for East (E), North (N) and Vertical (h) directions.
3. “red-light” or warning level (code 3). In the case of Madonna del Sasso, the level “red light” was never reached in E and N directions. The anomalous data (4%) for the vertical component (h) after a control were not validated for a processing problem.

#### 4.2.2. Alert Assessment Based over a Long-Term Period Trend

Another automatic control made by the algorithm is searching for anomalous acceleration trends in a long-term period. In this case, an alarm must be provided if the displacement rate, calculated from the linear regression, exceeded fixed ST and GT.

After having computed the parameters of the trend line, the statistical significance of the velocity is calculated using a probability value setting parametrically. This value is set at 90%, and this means that the measurement must be with low noise from the regression line to avoid to have spike measure.

The software procedure, developed in Matlab language, also calculates the absolute value of this speed, using as an index the number of millimeters of displacement every hundred theoretical days of measurement.

In addition, in this case, a vector of three components is written in an output file and, for each baseline, indicates with zero the non-significativity of the estimate speeds. A value not equal to zero, with the sign, indicates that the speed in mm/100 days on that coordinate. Any speed that, in absolute value, does not exceed 3 mm/100 days is considered non-significative, based on the results of topographic monitoring and Colombero studies. In addition, this parameter and the probability value of significance are variable parametric indices.

The preliminary results of GNSS monitoring have too short temporal time series to make a reliable analysis. In further studies, it would be possible to analyze the entire seasonal cycle.

**Figure 11:**
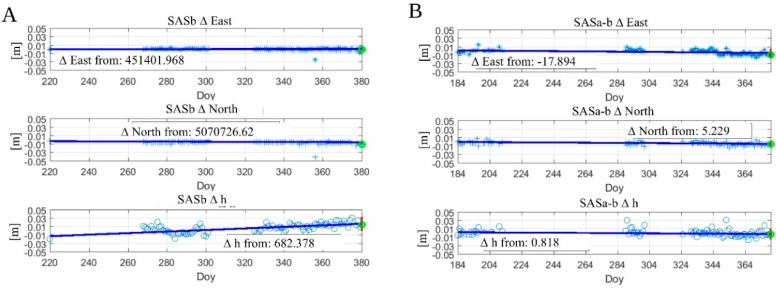
An example of displacement and deformation time series output sent to a monitoring expert. (**A**) computation of the E, N, and h displacements from the difference with original WGS84 32N coordinates for the GNSS SAS b; (**B**) computation of the deformation calculated from the original distance between the GNSS SAS a and SAS b. The time is expressed in Doy (Day of the year, where 0 = is 1 January 2018).

Figure 11 shows an example of the daily automatic output of GNSS processing: Figure 11A shows graphs of displacement time series of one of the two stations of Madonna del Sasso (SAS b). In Figure 9B, we see the time series of the deformations. For deformation, we intend the difference of displacement between the two stations, both placed on the Madonna del Sasso landslide (SASa-b).

All the graphs are also located daily in the shared Google Drive directory and a daily email that contains the link to a shared cloud directory and sends an ASCII file of the “traffic light” synthesis to geologists specialized in deformation analysis.

The short-term analysis of displacement did not show critical acceleration, and the movement is all inside the noise (2σ) of the measurements. Also, the short-term GT was not yet defined as the more extended time series of data is necessary.

Even if the long-term displacement has too short temporal series (less than half seasonal cycle) to make reliable interpretation of data, we try to make a preliminary analysis of available data (December 2018–June 2019) and a comparison with ARPA Piemonte topographic measurements (T2 benchmark). Unfortunately, lighting damages limited the possibility to have a more extended and continuous GNSS time series.

**Figure 12:**
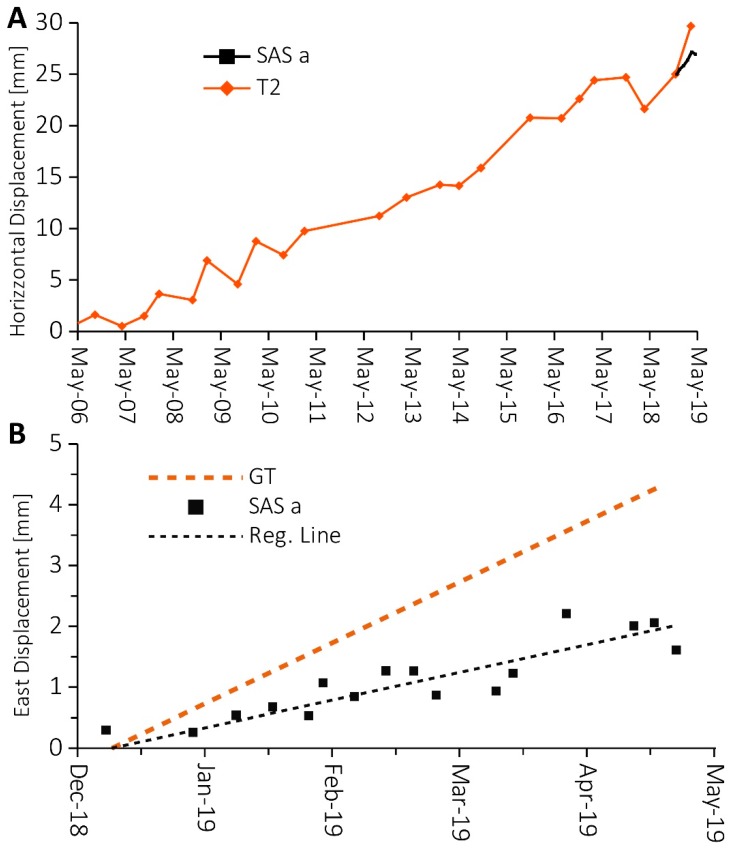
(**A**) shows a comparison of horizontal displacement between GNNS “SAS a” based on weekly averaged measurements from December 2018 to May 2019 and topographic “T2” for the period 2006–2019; (**B**) shows GNSS “SAS a” East displacement averaged with weekly sampling and its regression line (equation f_(x)_ = 0.01x − 634.47; R^2^ = 0.86) compared with geological thresholds (GT) of 3 mm/100 days (11 mm/year).

In the figure (Figure 12A), it is possible to see that the period of continuous GNSS measures only one interval between two topographic measures. Despite the short period of measure, the magnitude of planimetric displacement is similar.

If we focus the analysis on the period of GNSS continues acquisition (Figure 12B), it is possible to see that, if the displacement is averaged on a weekly window, the time series shows low noise (the correlation coefficient, R^2^ = 0.86). This sampling interval of seven days is based on an empirical test that, for this rate of deformation, best fit acceptable noise with measure frequency. The results show that the rate of horizontal displacement calculates with linear regression is about 4.6 mm/year, below the GT of 3 mm in 100 days (≈11 mm/year).

## 5. Discussion

In this paper, we present the application of a low-cost GNSS system for continuous monitoring of the Madonna del Sasso rock cliff. In the last few decades, different authors studied the dynamic of this slope instability that nowadays is quite known. According to the kinematic model of the slope, ARPA decided to check the evolution of the two unstable blocks by six-monthly topographic campaigns. This control of displacement can be considered enough for checking the defined trend or for the detection of changes in the long-term deformation rate. The limit of this low-frequency monitoring solution is the possibility that the slope could change his behavior immediately after a measurement and a possible dangerous evolution of the instability cannot be detected soon. On the other hand, traditional continuous monitoring systems are not affordable.

For this reason, a possible solution could be the use of continuous low-cost GNSS. The use of low-cost GNSS is characterized by pros and cons that can be synthetized as follows:

Among the advantages, we can list:

- With an adequate processing of the data and correct installation of the instrument, the accuracy and precision of the low-cost instrument are almost the same (5 mm in planimetry and 10 mm in altimetry) of traditional GNSS.
- The continuous and high frequency of measure could be able, over a long-term period of monitoring (some seasonal cycles), to better describe the process of expansion-contraction of the rock mass according to geophysical studies [22].
- The use of continues monitoring solution also allows the early detection of changes in displacement trend that could be indicative of a possible slope failure. This early warning procedure is based not only on the use of GNSS sensors but on the development of an automatized procedure that can acquire, process, and check if the displacement rate is higher than predefined thresholds.
- This procedure is fully automatized and can work without human intervention.

On the other hand, this experimental system showed some limitations:

- The continuity of measure and the maintenance of the system is fundamental, to have measurements’ consistency when a system became operative. In our study, some problems related to lighting and benchmark repositioning have limited the period of observation (22% of days without alarm estimation). However, it is to take into account the experimental condition of our research.
- Some problem GNSS constellations: The Beidou constellation did not yet have a sufficient number of satellites to improve the robustness of the positioning. The Galileo constellation, which is more visible in Europe, was not yet complete in late 2018. Despite this, we have chosen to use these three constellations because of the future and rapid improvements in both the constellations and the processing software used.
- Even if it is a low-cost solution, the required efforts are similar or higher efforts available for periodical measurements.
- The slow-moving process needs a large time series to be understood even with continuous monitoring. This means that, if the entity of displacement is close to the accuracy of the system, the first sessions that show a trend change can be considered spikes. Only after a week can we be sure that a change in the displacement trend can be really identified and certified.

## 6. Conclusions

In this paper, we propose not only a technical solution that adopts mass-market instrumentation, but also the definition of a monitoring systems management strategy that can be adopted for GNSS taking into consideration different phases: analysis, validation, interpretation, and dissemination of obtained results.

We tested the potentiality of low-cost GNSS for continuous monitoring of unstable slope and we propose a step by step methodology for semi-automatic alert messages based on continuous monitoring data.

The unstable slope affecting Madonna del Sasso sanctuary represents a good case study of this scenario. According to ARPA monitoring system results, we know that the unstable sector of this area site is characterized by an annual displacement trend of 2 mm/year. The importance of this cultural heritage site has prompted in the last few decades several research groups to study the slope instability with different approaches. Nowadays, there is an important dataset that can be used for the characterization of the slope and of displacement trends. We collect and analyzed the primary outcomes of literature studies and the analysis of the non-continuous monitoring system of ARPA Piemonte in an Operative Monography, a document that helped us in the GNSS system planning and installation. The ARPA monitoring network can be considered a suitable solution for the control of the evolution of the studied area. The semiannual frequency of measurement campaigns, however, is not sufficient to detect a rapid acceleration related potentially related to a paroxysm slope instability.

The use of the proposed GNSS low-cost monitoring network can be considered an affordable solution to improve the acquisition rate. The installed low-cost GNSS receivers have shown similar performance in terms of accuracy and precision respect geodetic GNSS on the order of a few millimeters in laboratory tests ([15]. Considering that the cost is ten times lower, the precision does not decreases significantly. With the completion of the new satellite constellations (e.g., Galileo), the improvement of the processing hardware and software can be expected to reduce costs and increase monitoring accuracy.

We also implemented a methodology that is based on a semi-automated procedure able to control the correct running of the system and the quality of results. In the first step, an automatized procedure checks if the obtained results are statistically correct. In the second step, the system checks if the displacement trend exceeded predefined thresholds and output light-traffic alert system. The final check done by experts that validate alert messages before publication: it is necessary to have reliable data, especially when the geological model of the unstable slope is not well known. The last phase of the proposed methodology also considers the emission of the alert bulletin.

The first period of monitoring was used to test the automatic alarm thresholds, and show that the system is run adequately filtering very noised measurements and false alarms. However, further studies are needed to asses a reliable critical geological threshold on a short time interval.

The first partial results on the long-term the trend of displacement registered by GNSS shows that it is comparable with topographic data in the same interval (≈5 mm/year). With a sampling interval of a week, the rate of displacement can be calculated with millimetric precision. In the future, with more extended time series, it would be possible to understand better the behavior of unstable blocks.

The results of this study show that the GNSS low-cost solution can be particularly interesting when we have to control the evolution of low rate slope instabilities that could be affected by a sudden increment of displacement that can be considered the precursory of a failure. It can also help to install a more precise and expansive monitoring system only during emergency phases. This approach has the aim to make affordable the constant monitoring of many sites today monitored only with discontinues instrumentation.

## Figures and Tables

**Table 1:** List of ARPA Piemonte monitoring data used for this work.

CODE	Name	Type	from–to	Active
L7MDSA0	D1	Laser distance meters	2009–2018	yes
L7MDSA1	D2	Laser distance meters	2009–2018	yes
T7MDSA3	T0	Topographic Benchmark	2006–2019	yes
T7MDSA2	T1	Topographic Benchmark	2006–2019	yes
T7MDSA1	T2	Topographic Benchmark	2006–2019	yes
T7MDSA0	T3	Topographic Benchmark	2006–2019	yes
AO7ND	PS1	InSAR data (RADARSAT)	2003–2009	no

**Table 2:** Madonna del Sasso site. The unit cost of instrumentations and the estimate installation cost.

Instrument	Unit Cost
GNSS uBlox EVK8T Receivers	70 €
Garmin antenna	50 €
Tri-axial accelerometer + temperature sensor	150 €
Micro PC mainboard, plastic box and dongle Wi-Fi	200 €
Installation cost	≈500 €

**Table 3:** The Ratio test (R) threshold, the statistical (ST), and the geological (GT) thresholds used for a Madonna del Sasso case study.

Displacement Type	R	ST	GT
Short term (Hourly to daily)	≥4	≥2σ	Not yet defined
Long term (Monthly to semester)	≥4	≥2σ	>3 mm/100 days (≈11 mm/year)

**Table 4:** Displacement rate, azimuth, and dip measured by topographic benchmark, InSAR and laser distance meter monitoring of ARPA Piemonte (measured along the line of sight—LOS).

Instrument	Topographic Benchmarks				Laser Distance	InSAR
Name	T0	T3	T2	T1	D2	Radarsat PS_VLOS_
Temporal Span [Year]	2006–2019				2009–2018	2003–2009
Vel_xy_ [ mm/year]	1.4	1.2	2.1	1.7	-	
Azimuth [°]	55	73	55	64	-	80 _LOS_
Vel_xyz_ mm/year	2.9	2.6	3.2	3.4	2.7 _(LOS)_	2.9 _LOS_
Dip[°]	61	63	50	58	-	56 _LOS_

**Table 5:** The alarm code for East, North, and vertical components the for the period December 2018–May 2019 for the GNSS SAS a.

Alarm Color	Alarm Code	Number of Alarm			% of Alarm			% of Fixed Alarm		
		E	N	h	E	N	h	E	N	h
Not Fixed	0	37	37	37	22%	22%	22%	-	-	-
Green	1	127	127	120	77%	77%	72%	98.5%	98.5%	93.0%
Yellow	2	2	2	2	1%	1%	1%	1.5%	1.5%	1.5%
Red	3	0	0	(7)	0%	0%	(4%)	0%	0%	(5.5%)
Validated	-	-	-	No	-	-	No	-	-	No
